# Folic Acid-Functionalized Black Phosphorus Quantum Dots for Targeted Chemo-Photothermal Combination Cancer Therapy

**DOI:** 10.3390/pharmaceutics11050242

**Published:** 2019-05-21

**Authors:** Miaomiao Luo, Wei Cheng, Xiaowei Zeng, Lin Mei, Gan Liu, Wenbin Deng

**Affiliations:** School of Pharmaceutical Sciences (Shenzhen), Sun Yat-sen University, Guangzhou 510275, China; luomm3@mail2.sysu.edu.cn (M.L.); upc201412@163.com (W.C.); zengxw23@mail.sysu.edu.cn (X.Z.); meilin7@mail.sysu.edu.cn (L.M.); dengwb5@mail.sysu.edu.cn (W.D.)

**Keywords:** cancer targeted therapy, drug delivery, black phosphorus, quantum dots, photothermal therapy

## Abstract

Due to the inherent limitations, single chemo or photothermal therapies (PTT) are always inefficient. The combination of chemotherapy and PTT for the treatment of cancers has attracted a great interest during the past few years. As a photothermal agent, black phosphorus quantum dots (BPQDs) possess an excellent extinction coefficient, high photothermal conversion efficacy, and good biocompatibility. Herein, we developed a photo- and pH-sensitive nanoparticle based on BPQDs for targeted chemo-photothermal therapy. Doxorubicin (DOX) was employed as a model drug. This nanosystem displayed outstanding photothermal performance both in vitro and in vivo. Folic acid conjugation onto the surface endowed this system an excellent tumor-targeting effect, which was demonstrated by the cellular targeting assay. The BPQDs-based drug delivery system exhibited pH- and photo-responsive release properties, which could reduce the potential damage to normal cells. The in vitro cell viability study showed a synergistic effect in suppressing cancer cell proliferation. Therefore, this BPQDs-based drug delivery system has substantial potential for future clinical applications.

## 1. Introduction

Nowadays, cancer has become one of the deadliest diseases around the world [1]. A number of clinical treatments have been developed for cancer therapy, such as surgery, chemotherapy, immunotherapy, radiotherapy, photodynamic therapy (PDT), and photothermal therapy (PTT) [2,3,4,5,6]. Compared to others, chemotherapy is still considered as the dominant medical modality [2,7]. However, chemotherapy is often not very efficient and sometimes fails to kill cancer owing to low accumulation of chemotherapeutic drugs in tumor sites [1,8]. As a noninvasive and harmless therapeutic alternative or supplement to traditional cancer therapy, PTT has gained considerable attention in recent years because of its higher selectivity and lower side effects [9,10]. However, as a matter of fact, both chemo and photothermal therapies have their own limitations [11]. Thus, the combined treatment of chemotherapy and PTT has attracted a great interest during the past few years [11,12]. The synergistic effect of chemo-photothermal therapy is expected to cooperatively suppress cancer development and improve the cancer treatment effect [13,14].

The ideal near infrared (NIR) laser absorbing photothermal agent should have a considerable extinction coefficient as well as high photothermal conversion efficacy in the NIR region [15,16]. To date, a variety of PTT agents have been well explored, such as noble metal-based nanostructures, semiconductor nanoparticles, and carbon-based nanomaterials [17,18,19,20,21]. As a new two-dimensional (2D) material, black phosphorus (BP), a metal-free layered semiconductor, has a layer-dependent bandgap of 0.3 eV (bulk materials) to 2.0 eV (monolayered BP), resulting in a broad absorption across the ultraviolet and infrared regions [22,23]. BP quantum dots (BPQDs), the ultrasmall derivatives of BP nanosheets, possess an excellent extinction coefficient, high photothermal conversion efficacy, and high specific surface area [15,22], which endow it with the capacities for photothermal therapy and drug delivery [24]. As ideal photothermal therapy (PTT) gents, BPQDs fully satisfy the strict safety requirements in clinical use, because P is a vital element in human bodies (accounting for about 1% of the total body weight) [22,25]. Furthermore, BPQPs can be degraded into nontoxic and biocompatible phosphorus oxides (phosphate or phosphonate) in vivo, which can be well tolerated by the human body [25,26]. To the best of our knowledge, using BPQDs as a drug delivery platform has been investigated rarely.

**Figure 1:**
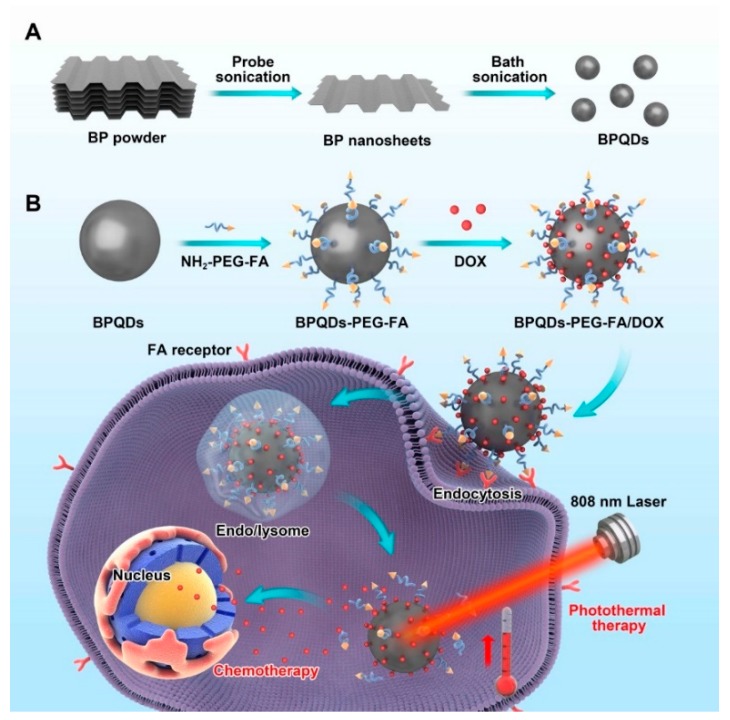
Schematic depiction of preparing black phosphorus quantum dots (BPQDs)-PEG-FA/DOX (doxorubicin) and their in vitro combined chemo-photothermal therapy. (**A**) Schematic illustration of the preparation of BPQDs; (**B**) Schematic illustration of BPQDs-based drug delivery system for synergistic photothermal/chemotherapy of cancer.

Herein, we designed a BPQDs-based drug delivery nanosystem for synergistic actively targeted chemo-photothermal therapy (Figure 1A) and applied this nanoplatform in cancer theranostics. BPQDs were obtained by a modified liquid exfoliation method (probe sonication followed by bath sonication of BP powder). Then, to endow BPQDs with active targeting functionality, folic acid (FA), a B vitamin with specific tumor-targeting properties [27,28,29], was conjugated on the surface of BPQDs by electrostatic interactions (Figure 1B) [30]. Subsequently, the folic acid-functionalized BPQDs were loaded with anticancer drugs (doxorubicin, DOX) for chemotherapy. As expected, this drug delivery system exhibited enhanced cancer cell killing ability, benefiting from the synergistic combination of chemo-photothermal therapy.

## 2. Experimental Section

### 2.1. Materials

The bulk BP was purchased from Nanjing muke nano technology co. LTD (Nanjing, China). N-methyl-2-pyrrolidone (NMP), PEG-NH_2_, FA-PEG-NH_2_, 3-(4,5-dimethyl-2-thiazolyl)-2,5-diphenyl-2-H-tetrazolium bromide (MTT), 4′,6-diamidino-2-phenylindole (DAPI) and Dimethyl sulfoxide (DMSO) were all purchased from Sigma-Aldrich (St. Louis, MO, USA). Doxorubicin hydrochloride (DOX) was bought from Dalian Meilun Biology Technology Co., Ltd. (Dalian, China). Sodium hydroxide (NaOH) was purchased from Aladdin Industrial Co., Ltd. (Shanghai, China). All other chemicals and reagents of the highest quality were commercially available and used as received. Human cervical cancer cell line, human lung adenocarcinoma cell line, human breast cancer cell line, and human liver cancer line were bought from American Type Culture Collection (ATCC, Rockville, MD, USA). The foetal bovine serum, H-DMEM, RPMI 1640, phosphate buffer saline (PBS) (pH 7.4), penicillin-streptomycin, trypsin-EDTA were obtained from Gibco Life Technologies (Thermo Fisher Scientific Inc, Waltham, MA, USA).

### 2.2. Preparation of Black Phosphorus Quantum Dots (BPQDs)

An amount of 10 mg of bulk BP crystal powders were added into 10 mL of *N*-Methylpyrrolidone (NMP). The mixture was sonicated with a sonic tip in an ice bath for 6 h (amplifier: 25%, on/off cycle: 2 s/4 s). The mixture was then sonicated in water bath for 10 h. The resultant dispersion was centrifuged at a speed of 7000 rpm for 20 min. The supernatant was decanted gently and then centrifuged for 20 min at 12,000 rpm to obtain the precipitate (BPQDs).

### 2.3. Modification with PEG-FA

1 mg of the BPQDs were dispersed in 5 mL of H_2_O and then mixed with 5 mg of FA-PEG-NH_2_. After probe sonication for 30 min and stirring for 4 h, the mixture was centrifuged at 12,000 rpm and washed with water to remove excess FA-PEG-NH_2_ molecules. The product was designated as BPQDs-PEG-FA. PEG-modified BPQDs (BPQDs-PEG) were prepared using the same technology.

### 2.4. DOX Loading

BPQDs-PEG-FA (100 µg mL^−1^) was mixed with DOX aqueous solution (100 µg mL^−1^). The pH value of the mixture solution was adjusted to 8.5 using sodium hydroxide. After vigorous stirring in the dark for 2 h, the obtained DOX-loaded BPQDS (BPQDS-PEG-FA/DOX) were gathered by centrifugation and washed with water. The quantitation of DOX loading was obtained with UV−vis-NIR absorbance of DOX at 490 nm. The drug loading content (LC) was calculated as follows:

$$\mathrm{Drug}\;\mathrm{LC}\left(\%\right)=\frac{\mathrm{weight}\;\mathrm{of}\;\mathrm{drug}\;\mathrm{in}\;\mathrm{the}\;\mathrm{nanoparticles}}{\mathrm{weight}\;\mathrm{of}\;\mathrm{nanoparticles}\;\mathrm{taken}}\times 100\%$$

### 2.5. Characterization of BPQDs

Transmission electron microscopy (TEM) images were taken on the FEI Tecnai G 2 F30 transmission electron microscope (FEI Company, Hillsborough, OR, USA) at an acceleration voltage of 300 kV. Atomic force microscopy (AFM) was performed on an atomic force microscope (Bruker Dimension® Icon™, Karlsruhe, Germany) to characterize the height of the samples. The zeta potential of nanoparticles (NPs) was determined by Malvern Mastersizer 2000 (Zetasizer Nano ZS90, Malvern Instruments Ltd., Malvern, UK). Fourier transform infrared spectra (FT-IR) of the samples were obtained with a Thermo Scientific Nicolet iS 50 spectrometer (Thermo Fisher Scientific Inc, Waltham, MA, USA) using the KBr method. X-ray photoelectron spectroscopy (XPS) was carried out using an X-ray photoelectron spectroscope (Axis HSi, Kratos Ltd., Mancheste, UK) with Al Kα radiation (1486.6 eV photons, 150 W) as the X-ray source for excitation. A high-resolution confocal Raman microscope (HORIBA LabRAM HR800, HORIBA Scientific, Paris, France) was employed to obtain Raman spectra. UV−vis-NIR absorption spectra were recorded by an Infinite M200 Pro TECAN GENIOS (Tecan Group, Männedorf, Switzerland).

### 2.6. Photothermal Effect of BPQDs-PEG-FA

The photothermal heating effects of different samples (water, BPQDs, BPQDs-PEG-FA) were determined by measuring the temperature changes of various solutions containing various concentrations of NPs (50−500 μg mL^−1^) under the irradiation of an 808 nm NIR laser (Shanxi Kaisite Electronic Technology Co., Ltd., Xi’an, China) with different laser power density (0.5–2.0 W cm^−2^). Real-time thermal imaging and the temperature change were recorded by an infrared thermal imaging camera (Ti450, Fluke, Everett, WA, USA).

### 2.7. pH and Photothermally-Triggered Drug Release Study

For the pH-responsive release, 1 mL of BPQDs-PEG-FA/DOX was sealed in a dialysis bag (MWCO = 3500, Shanghai Sangon, Shanghai, China), and then immersed in 10 mL phosphate buffer saline (PBS)solutions (pH 7.4 or 5.0). At designated time points, 500 μL of outside release media was collected to measure the amount of DOX with an absorbance spectrometer, and 500 μL of fresh media was added. For the photo-responsive release, the study was performed at pH = 5.0 with an 808 nm NIR laser for 6 min at a power density of 1 W/cm^2^.

### 2.8. Cell Culture

HeLa, MCF-7, and HepG2 cells were cultured in Dulbecco’s modified eagle medium (DMEM) supplemented with 10% fetal bovine serum (FBS) and 1% penicillin-streptomycin in a humidity atmosphere of 5% CO_2_ at 37 °C. A549 cells were cultured in the Gibco® RPMI 1640’s culture medium containing 10% fetal bovine serum (FBS) and 1% penicillin-streptomycin.

### 2.9. In Vitro Cellular Uptake Assays

HeLa cells were seeded on 20 mm glass-bottom Petri dishes and cultured overnight. The cells were then treated with DOX, BPQDs-PEG/DOX, and BPQDs-PEG-FA/DOX (DOX concentration 5 μg/mL) for 0.5 and 2 h. At indicated time points, cell medium was removed, and cells were washed with PBS, followed by fixing with 4% paraformaldehyde for 20 min and washing with PBS. The fluorescence intensity was detected using a confocal laser scanning microscope (CLSM, Olympus Fluoview FV-1000, Tokyo, Japan).

The quantitative analysis was performed by flow cytometry. Briefly, HeLa cells were incubated with DOX, BPQDs-PEG/DOX, BPQDs-PEG-FA/DOX, and BPQDs-PEG-FA/DOX + free folic acid (5 μg/mL DOX) for 1 h. Then the cells were washed with PBS and collected. The intracellular fluorescence of DOX was detected by FCM (BD Biosciences, San Jose, CA, USA) at an excitation wavelength of 490 nm.

### 2.10. In Vitro Cytotoxicity Assays

HeLa cells were seeded on 96-well plates at a density of 5 × 10^4^ cells per well and cultured overnight. The old cell culture medium was replaced with fresh DMEM medium containing different NPs at various concentrations (0.125–2.5 μg DOX/mL). After incubation for 24 or 48 h, 10 μL of MTT solution in medium (5 mg mL^−1^) was added into each well and incubated for another 4 h. Then the mixture was removed, and 100 μL of DMSO was added. After gentle shaking for 20 min, the absorbance was measured using a microplate reader at a wavelength of 490 nm. The cell viability was normalized to the control group without any treatment. Similar experiments were carried out on MCF-7, A549, and HepG2 cells.

### 2.11. In Vitro Photothermal Therapy Study

HeLa cells were seeded in a 96-well plate and cultured overnight. BPQDs, BPQDs-PEG, and BPQDs-PEG-FA were then added to the cells with different concentrations. After incubation for 4 h, the cells were irradiated with 808 nm NIR laser for 10 min and incubated for another 12 h. Subsequently, the cell viability was evaluated by MTT assay.

### 2.12. Xenograft Tumor Models

The protocols for animal assays were approved by the Administrative Committee on Animal Research in Sun Yat-sen University. Guidelines of the institutional animal ethics committee were followed for in vivo experiments. The project identification code is SYSU-IACUC-2018-B3072 and the date is December 2nd 2018). Four- to five-week-old female severe combined immunodeficient (SCID) mice were obtained from the Guangdong Medical Laboratory Animal Center. 100 µL of HeLa cell suspension in PBS (~2 × 10^6^ cells) was injected subcutaneously on the dorsal side of the mice to induce tumors.

### 2.13. In Vivo Photothermal Images

HeLa tumor-bearing nude mice were injected with 100 μL of PBS, BPQDs-PEG/DOX, and BPQDs-PEG-FA/DOX suspension. Twelve hours after injection, the tumor-bearing mice were irradiated with 808 nm NIR laser at a power density of 1.5 W cm^−2^ for 5 min. The tumor temperature change was recorded by an infrared thermal imaging camera.

### 2.14. In Vivo Combined Therapy

When the tumor size reached about 200 mm^3^, the HeLa tumor-bearing mice were intraperitoneally injected with saline, free DOX, BPQDs-PEG/DOX, BPQDs-PEG-FA/DOX, and BPQDs-PEG-FA/DOX with NIR laser irradiation, respectively, at a dose of 5 mg DOX kg^−1^ in PBS twice a week. Tumors and body weight were measured every other day. The volume (V) of the tumor was calculated as follows: *V* = *d*^2^ × *D*/2, where *D* and *d* represent the longest and shortest diameters of the tumor, respectively.

### 2.15. Statistical Methodology

Unless stated otherwise, all the experiments were carried out at least three times. The experimental data are expressed as mean ± standard deviation (SD). Statistical analysis was performed by one-way ANOVA followed by Bonferroni test with SPSS 22.0 software (IBM, Chicago, IL, USA, 2011). * *p* < 0.05 as statistical significance and ** *p* < 0.01 as extreme statistical significance.

## 3. Results and Discussion

### 3.1. Characterizations of the Drug Delivery Platform

**Figure 2:**
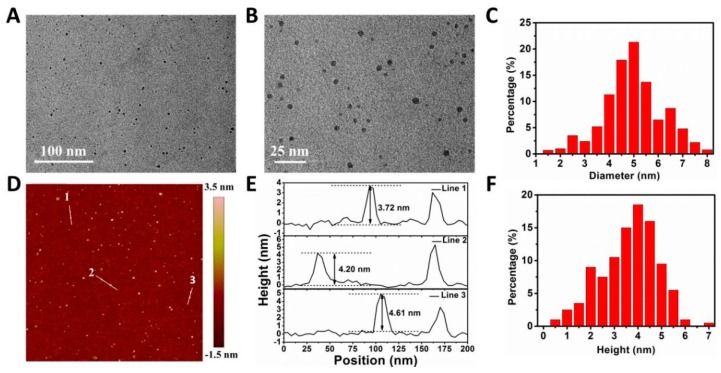
Characterizations of the NPs. (**A**) Transmission electron microscopy (TEM) image. (**B**) Magnified TEM image. (**C**) Statistical analysis of the size of 100 BPQDs-PEG-FA/DOX according to the TEM images. (**D**) Atomic force microscopy (AFM) image of BPQDs-PEG-FA/DOX. (**E**) Height profiles along the white lines in D. (**F**) Statistical analysis of the size of 100 BPQDs-PEG-FA/DOX based on the AFM images.

The morphology of nanoparticles (NPs) was revealed by transmission electron microscopy (TEM) and atomic force microscopy (AFM). As shown by TEM images (Figure S1), BPQDs had a uniform shape with an average lateral size of 2.8 ± 0.7 nm. The AFM images (Figure S2) showed the topographic morphology of BPQDs. The average height of BPQDs was 1.8 ± 0.6 nm. As displayed in Figure 2A–F, compared with BPQDs, BPQDs-PEG-FA/DOX possessed a larger size (4.9 ± 1.0 nm) and greater height (3.8 ± 0.9 nm), demonstrating the successful coating of FA and DOX molecules. The successful preparation of BPQDs-PEG-FA/DOX could also be proved by the surface charge analysis (Figure S3), in which the zeta potential increased from −25.6 mV (BPQDs) to −8.4 mV (BPQDs-PEG-FA/DOX).

**Figure 3:**
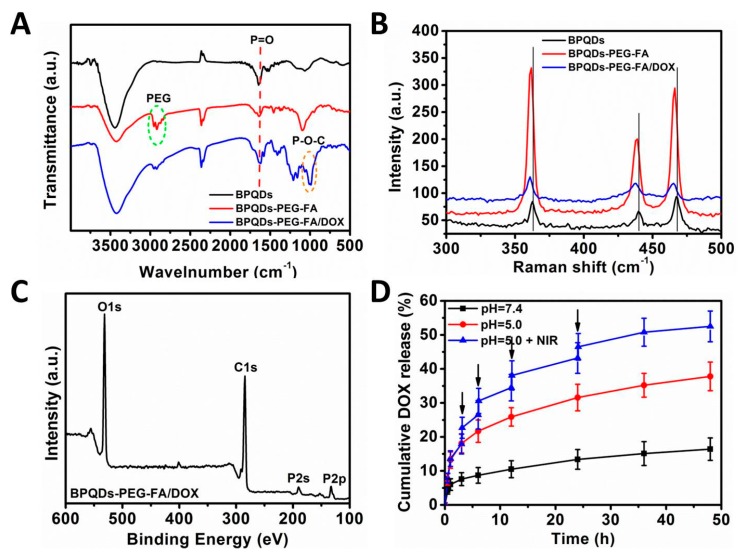
Characterizations of the nanoparticles (NPs). (**A**) Fourier transform infrared (FT-IR) spectrum of the BPQDs-based nanoparticles. (**B**) Raman spectra of the NPs. (**C**) X-ray photoelectron spectroscopy (XPS) spectrum of BPQDs-PEG-FA/DOX. (**D**) DOX release profiles of BPQDs-PEG-FA/DOX at different pH values with or without near infrared laser irradiation (1.0 W cm^−2^, 6 min).

Figure 3A presents the FT-IR spectra of BPQDs, BPQDs-PEG-FA, and BPQDs-PEG-FA/DOX. The peak at ~1630 cm^−1^ for all samples was ascribed to the P=O stretching mode [31,32]. The absorption bands centered at ~2900 cm^−1^ of BPQDs-PEG-FA could be assigned to the successful conjugation of PEG-FA [30]. After DOX loading onto the BPQDs, several new peaks appeared. The absorbance peak at ~1000 cm^−1^ was due to the formation of P–O–C bonds [32], indicating that DOX molecules were chemically bound with BPQDs through P–O–C bonds. The NPs were also characterized by Raman spectroscopy (Figure 3B). BPQDs exhibited three prominent peaks, which were ascribed to one out-of-plane phonon mode at 361.6 cm^−1^ ($A_{g}^{1}$) and two in-plane modes at 438.7 ($B_{2g}$) and 467.3 cm^−1^ ($A_{g}^{2}$) [33,34]. After the PEG-FA modification and DOX loading, blue-shifts were observed, which might be due to the slightly increased height of the BPQDs. The nanoparticles were further characterized by X-ray photoelectron spectroscopy (XPS). Compared with BPQDs and BPQDs-PEG-FA (Figure S4), the peak intensity of P in BPQDs-PEG-FA/DOX was much weaker (Figure 3C), indicating that a thin layer of molecules (DOX) was coated onto the surface of BPQDs-PEG-FA. Furthermore, the strong intensity of C in BPQDs was due to the surface adsorption of C which was used for calibration.

### 3.2. Drug Loading Capacity and Release Behavior Study

Then, we tested the drug loading capacity and drug release behavior of BPQDs­PEG­FA. The DOX loading capacity of BPQDs­PEG­FA increased as DOX/NPs feeding ratio increased (Figures S5 and S6). The DOX loading was measured to be ~160% when the feeding ratio reached 8, which was markedly higher than many conventional NPs-based drug delivery platforms (~10–30%) [30,35]. Figure 3D shows the release profiles of DOX under different pH values. At pH 7.4, only ~16.4% of DOX was released from BPQDs­PEG­FA/DOX, while ~37.8% of DOX was released at pH 5.0. This result was probably due to the acidic condition promoting the protonation of the amino group in DOX, while the physiological condition enhanced the electrostatic attraction between DOX and NPs [30,36]. Moreover, when the NPs were irradiated with a momentary NIR laser (6 min) for several times, a higher DOX release (52.5%) could be observed. These results demonstrated the pH-responsive and photothermally responsive drug release property of the BPQDs-based drug delivery nanoplatform, which could minimize the side effects of anticancer drugs and enhance antitumor efficacy.

### 3.3. In Vitro NIR Photothermal Performances

**Figure 4:**
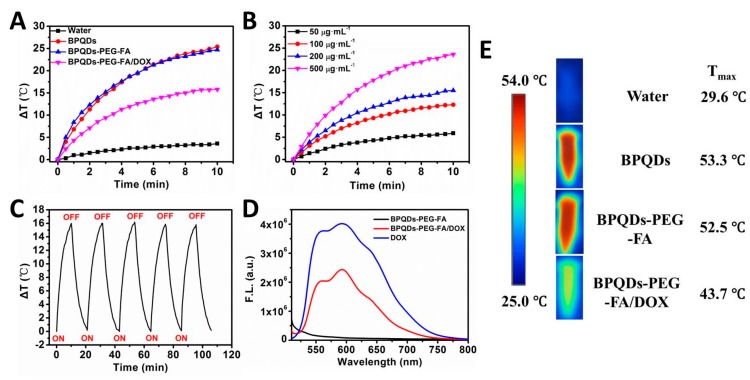
(**A**) Photothermal heating curves of pure water and BPQDs-based nanoparticles dispersed in water under irradiation with an 808 nm laser (200 μg BP mL^−1^, 1 W cm^−2^, 10 min,). (**B**) Temperature variation curves of the BPQDs-PEG-FA/DOX solution under different concentrations. (**C**) Heating of a suspension of the BPQDs-PEG-FA/DOX in water for five laser on/off cycles with an 808 nm NIR laser at power density of 1.0 W cm^−2^. (**D**) Fluorescent spectra of DOX (25.8 μg mL^−1^), BPQDs-PEG-FA (100 μg mL^−1^), and BPQDs-PEG-FA/DOX (25.8 μg mL^−1^ DOX, 100 μg mL^−1^ BPQDs-PEG-FA) aqueous solutions (λex = 490 nm). (**E**) NIR thermal images of water and BPQDs-based NPs under continuous NIR laser irradiation (1.0 W cm^-2^) for 10 min.

The NIR photothermal performances of the BPQDs-based NPs were investigated, and the results are displayed in Figure 4 and Figure S7. As displayed in Figure 4A,E, the temperature of the BPQDs, BPQDs-PEG-FA, and BPQDs-PEG-FA/DOX solution increased by 25.4, 24.7 and 15.8 °C, respectively (1 W cm^−2^, 10 min, 200 μg BP mL^−1^), which was much higher than that of water (Δ*T* ≈ 3.6 °C). As shown in Figure 4B and Figure S7, a concentration- and power intensity-dependent photothermal effect of BPQDs-PEG-FA/DOX was observed. A similar photothermal heating effect of BPQDs could also be found (Figure S8). Furthermore, the photothermal stability of BPQDs-PEG-FA/DOX was evaluated (Figure 4C). During the process of continuous laser irradiation for 5 cycles, no significant temperature change was observed, suggesting the excellent photothermal stability of BPQDs-PEG-FA/DOX. Moreover, we performed a fluorescence quenching experiment (Figure 4D). The fluorescence intensity of loaded drug molecules could be partially quenched by folic acid-modified BPQDs, indicating the strong interaction between DOX molecules and BPQDs­PEG-FA [37].

### 3.4. In Vitro Cellular Uptake and Distribution Assay

**Figure 5:**
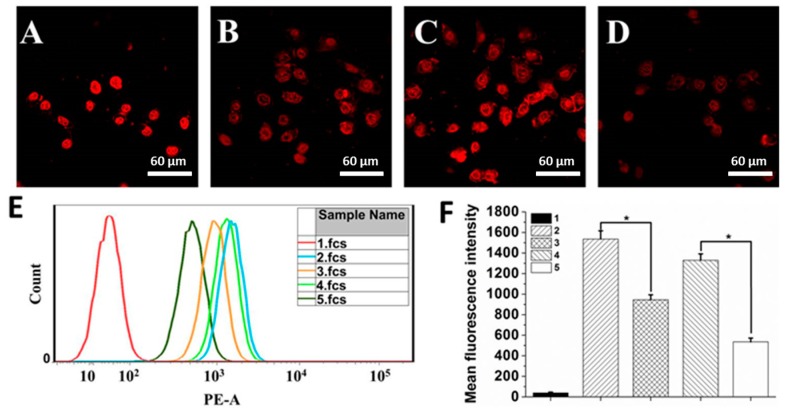
Confocal images of HeLa cells incubated with (**A**) free DOX, (**B**) BPQDs-PEG/DOX, (**C**) BPQDs-PEG-FA/DOX and (**D**) BPQDs-PEG-FA/DOX + free folic acid after incubation for 2 h. (**E**) Flow cytometry (FCM) histogram profiles of cellular DOX fluorescence intensities in HeLa cells after 1 h incubation. (**F**) Quantification analysis of DOX fluorescence intensity in HeLa cells after incubation for 1 h. 1, 2, 3, 4, and 5 represent control, DOX, BPQDs-PEG/DOX, BPQDs-PEG-FA/DOX, and BPQDs-PEG-FA/DOX + free folic acid, respectively. (* *p* < 0.05).

In the next set of experiments, we studied the cellular uptake behavior and intracellular distribution of free or loaded DOX (Figure 5A–D and Figure S9). The DOX accumulation was time-dependent for all of the samples. After 0.5 h of incubation, the red fluorescence (DOX) could be observed for both the free DOX group and the DOX-loaded BPQDs group, indicating the successful internalization of these NPs. DOX fluorescence intensity in cells after 2 h of incubation with BPQDs­PEG­FA/DOX was significantly stronger than that of BPQDs­PEG/DOX, suggesting that conjugation of FA improved the binding and uptake of NPs to folate receptor (FR)-positive HeLa cells. The excellent tumor targeting effect of FA was also proved by a receptor competition experiment. The DOX fluorescence intensity significantly decreased when HeLa cells were incubated with BPQDs­PEG­FA/DOX and free folic acid at the same time. These results corresponded with the quantitative analysis of the flow cytometry (FCM) assay (Figure 5E,F). Moreover, we did a colocalization study (Figure S10), showing that BPQDs­PEG­FA/DOX merged well with the LysoTracker. This result indicated that BPQDs-PEG-FA/DOX was taken up by endocytosis. For the free DOX group, the red fluorescence mostly appeared in the nuclei, while that of BPQDs­PEG/DOX and BPQDs­PEG­FA/DOX was observed not only in the nuclei but also in the cytoplasm after incubation for 2 h, applying that DOX-loaded BPQDs were firstly taken up by cells through the endocytosis pathway and then localized in cellular compartments, and subsequently, the loaded DOX was gradually released and accumulated into the nuclei. 

### 3.5. In Vitro Combined Therapy Effect

**Figure 6:**
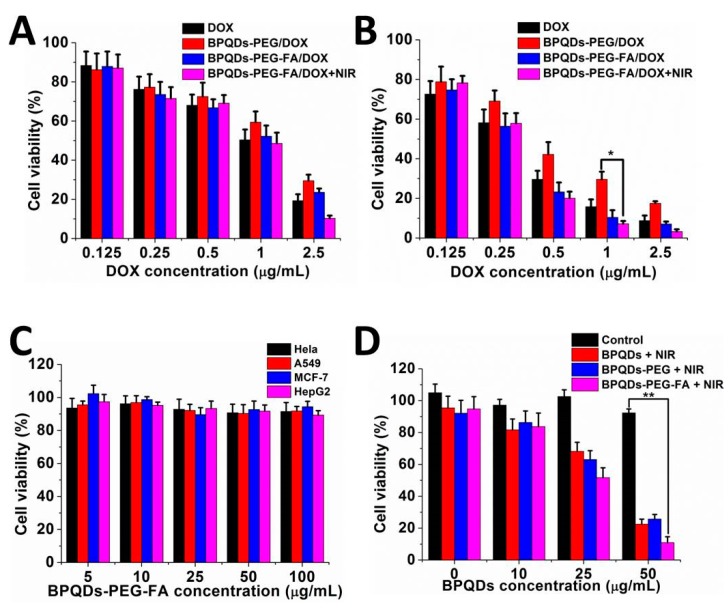
Relative viability of HeLa cells after treatment with DOX and different NPs for (**A**) 24 h and (**B**) 48 h. (**C**) Cell viability of HeLa, A549, MCF-7, and HepG2 cells with various concentrations of BPQDs-PEG-FA. (**D**) Relative cell viability of HeLa cells incubated with various concentrations of BPQDs-based NPs with NIR laser irradiation (808 nm, 1 W cm^−2^, 10 min). (* *p* < 0.05, ** *p* < 0.01).

We subsequently assessed the in vitro synergistic therapeutic effects of this BPQDs-based nanosystem as a combined chemo-photothermal agent. An MTT assay was performed to evaluate the cell viability. As shown in Figure 6A,B, the BPQDs-PEG-FA/DOX group exhibited a higher cytotoxicity compared with the free DOX and BPQDs-PEG/DOX groups after incubation for 48 h. This result might be ascribed to the relatively slower endocytosis of free DOX and non-targeted-nanoplatform. The BPQDs-PEG-FA/DOX with NIR irradiation group showed the highest cytotoxicity due to the synergistic effect. We next tested the potential ability of these nanoparticles as photothermal agents (Figure 6D). Excellent photothermal therapy efficiency of BPQDs and the BPQDs-based nanosystem in promoting cancer cell apoptosis could be observed. For example, around ~90% of HeLa cells were killed by BPQDS-PEG-FA (50 μg mL^−1^ BP) with an 808 NIR laser (1 W cm^−2^, 10 min), while negligible cytotoxicity was observed for the control group (cells without any NIR irradiation). Then, the biocompatibility of BPQDS-PEG-FA was investigated (Figure 6C). After 48 h of incubation, BPQDS-PEG-FA displayed no significant cytotoxicity to HeLa, A549, MCF-7, and HepG2 cells even at a relatively high concentration (100 μg mL^−1^). Taken together, the BPQDS-PEG-FA/DOX is very promising for the application in cancer treatment, due to the remarkable tumor targeting ability, excellent PTT effect, and especially low cytotoxicity.

### 3.6. In Vivo NIR Photothermal Performances

**Figure 7:**
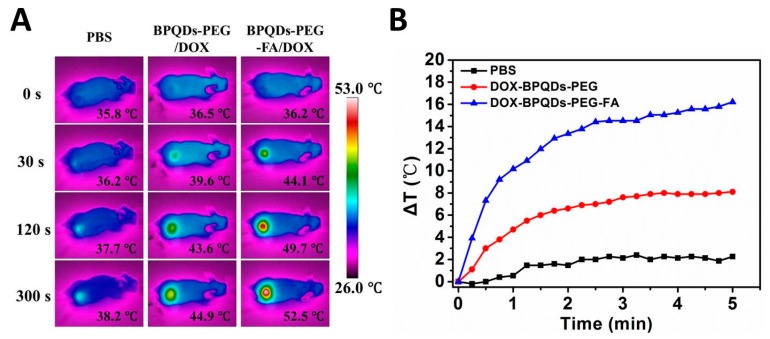
(**A**) Thermal images of HeLa tumor-bearing mice exposed to 808 nm laser (1.5 W cm^−2^) after injection of PBS, BPQDs-PEG/DOX, and BPQDs-PEG-FA/DOX, respectively. (**B**) Time-dependent temperature increase of HeLa tumor-bearing mice recorded by an IR camera under 808 nm laser (1.5 W cm^−2^).

Inspired by the exciting results in vitro, we further demonstrated the photothermal activity of the BPQDs-based drug delivery system in vivo. The results are shown in Figure 7A,B. For the BPQDS-PEG and BPQDS-PEG-FA treated groups, the temperature of the tumor sites in mice increased to 44.9 and 52.5 °C, respectively, within 5 min under the NIR irradiation, while the temperature of the tumor treated with PBS only increased to 38.2 °C. These results demonstrated the high efficiency of BPQDS-PEG-FA as a PTT agent and its excellent tumor targeting ability for in vivo tumor ablation.

### 3.7. In Vivo Combined Therapy Effect

**Figure 8:**
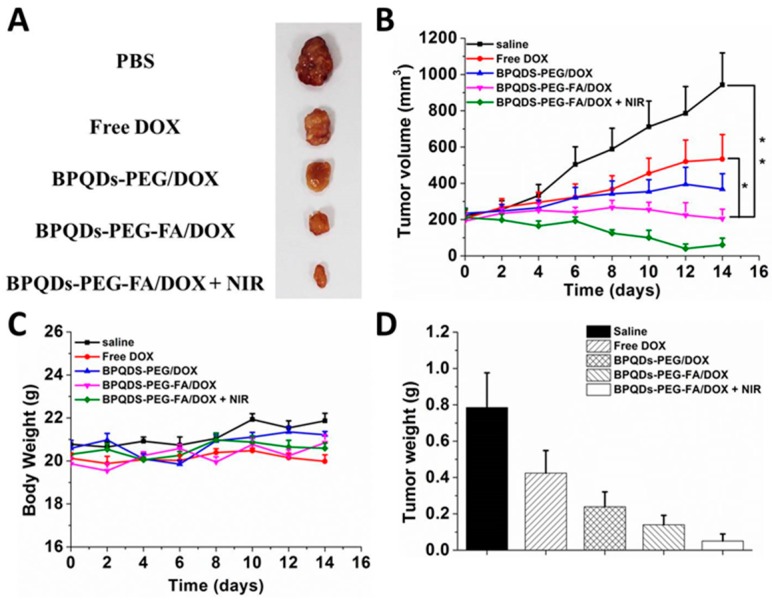
In vivo chemo-photothermal synergistic therapy. (**A**) Representative photos of excised tumors after 14 days of treatments. (**B**) Tumor growth curves of different groups of tumor-bearing mice after treatments. (**C**) The changes of body weight of tumor-bearing mice after treatments. (**D**) Tumor weight of each group taken out from the sacrificed mice at the end point of research (*n* = 3, * *p* < 0.05, ** *p* < 0.01).

In vivo studies were conducted to further evaluate the enhanced therapeutic effect of the chemo-photothermal combination therapy of BPQDS-PEG-FA/DOX (Figure 8). All four treatment groups (DOX, BPQDS-PEG/DOX, BPQDS-PEG-FA/DOX, and BPQDS-PEG-FA/DOX + NIR) showed active therapeutic effects, while the tumors of the control group were growing at a noticeable rate. As shown in Figure 8B, BPQDS-PEG/DOX could inhibit tumor growth to a certain extent, possibly due to longer retention of BPQDS-PEG/DOX in comparison with free DOX [38]. BPQDS-PEG-FA/DOX displayed a better tumor inhibition effect than BPQDS-PEG/DOX, which was attributed to the active tumor targeting effect of folic acid ligand, resulting in more accumulation of DOX. Remarkably, the tumor growth in mice treated with BPQDS-PEG-FA/DOX with NIR laser irradiation resulted in the best remission. Such an excellent antitumor ability could be ascribed to an enhanced specific delivery and outstanding synergistic effect of the combined chemo-photothermal therapy. Figure 8C shows the body weight curves of the treated mice throughout the whole treatment process.

## 4. Conclusions

In summary, we have successfully synthesized a therapeutic system based on BPQDs nanoparticles and demonstrated its promising application as a PTT agent and multifunctional drug delivery vehicle for the first time. This drug-loaded nanosystem displayed an outstanding in vitro antitumor therapeutic effect and no appreciable toxicity was observed from bare NPs. These folic acid-modified NPs achieved a high tumor targeting efficiency. The BPQDs-based drug delivery system exhibited pH- and photo-responsive release properties, which could reduce the potential damage to normal cells. Furthermore, the excellent photothermal performance of this system was also demonstrated in vivo. Thus, this BPQDs-based drug delivery system has substantial potential for future clinical application.

## Supplementary Materials

The following are available online at https://www.mdpi.com/1999-4923/11/5/242/s1, Figure S1: TEM images of (**A**), (**B**) BPQDs and (**C**), (**D**) BPQDs-PEG-FA, Figure S2: (**A**) AFM image of BPQDs. (**B**) Height profiles along the white lines in A. (**C**) AFM image of BPQDs-PEG-FA. (**D**) Height profiles along the white lines in C, Figure S3: Zeta potentials of BPQDs-based NPs. 1, 2, 3, 4, 5 and 6 represent BPQDs, BPQDs-PEG, BPQDs-PEG-FA, BPQDs-PEG/DOX and BPQDs-PEG-FA/DOX, BPQDs-PEG-FA at pH = 5 respectively, Figure S4: XPS spectrum of (**A**) BPQDs and (**B**) BPQDs-PEG-FA, Figure S5: DOX loading capacities on BPQDs-PEG-FA (*w*/*w* %) with increasing DOX/NPs feeding ratios, Figure S6: UV–vis–NIR spectra of BPQDs-PEG-FA /DOX at different DOX/NPs feeding ratios after the removal of excess free DOX, Figure S7: Photothermal heating curves of BPQDs-PEG-FA/DOX under different power intensities, Figure S8: Temperature variation curves of the BPQDs solution under different concentrations (1 W cm^−2^, 10 min), Figure S9: Confocal images of HeLa cells incubated with free DOX, BPQDs-PEG/DOX, BPQDs-PEG-FA/DOX and BPQDs-PEG-FA/DOX + free folic acid after incubation for 0.5 h, Figure S10: Confocal images of HeLa cells incubated with BPQDs-PEG-FA/DOX for 0.5 h and then were treated with Lyso-Tracker probes for 30 min. (**A**) DAPI; (**B**) BPQDs-PEG-FA/DOX; (**C**) LysoTracker; (**D**) merge.
