# Supplementary material for: Folic Acid-Functionalized Black Phosphorus Quantum Dots for Targeted Chemo-Photothermal Combination Cancer Therapy
Source: Pharmaceutics. 2019 May 21;11(5):242. doi: 10.3390/pharmaceutics11050242 (PMC6571808; doi:10.3390/pharmaceutics11050242)
Supplement: Supplementary file 1 [file pharmaceutics-11-00242-s001.pdf]

pharmaceutics

MDPI

Supplementary Materials: Folic Acid-Functionalized Black Phosphorus Quantum Dots for Targeted Chemo-Photothermal Combination Cancer Therapy

Miaomiao Luo, Wei Cheng, Xiaowei Zeng, Lin Mei, Gan Liu and Wenbin Deng

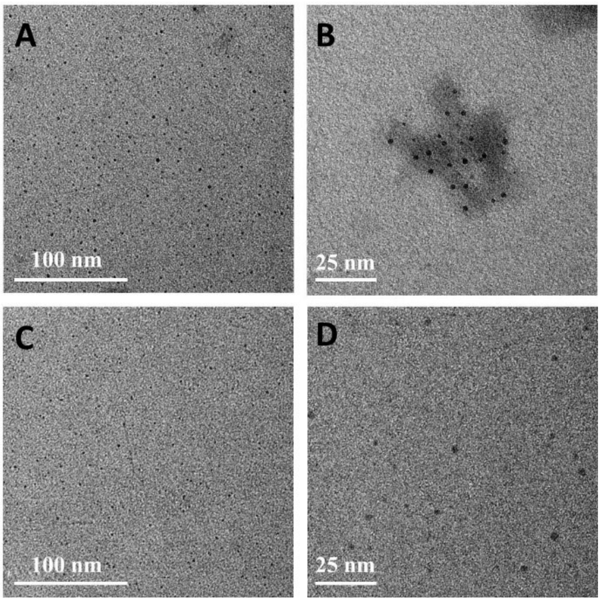

Figure 1. TEM images of (**A**)-(**B**) BPQDs and (**C**)-(**D**) BPQDs-PEG-FA.

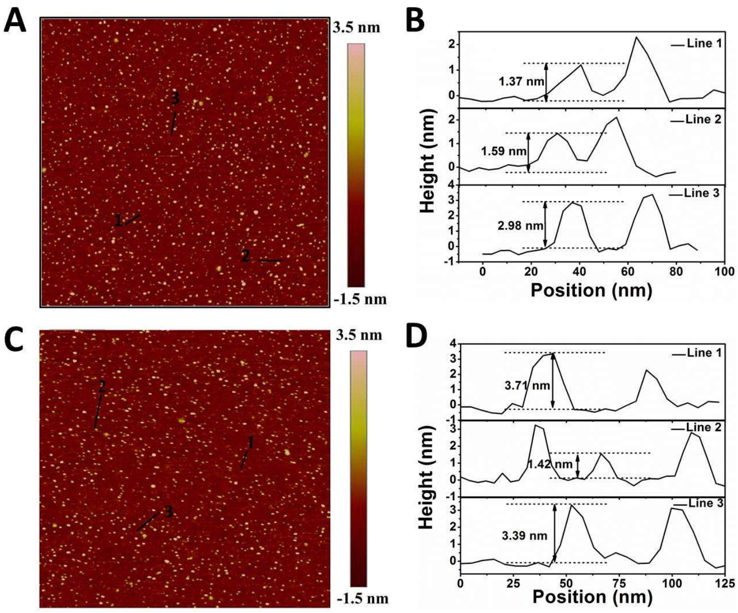

Figure 2. (**A**) AFM image of BPQDs. (**B**) Height profiles along the white lines in A. (**C**) AFM image of BPQDs-PEG-FA. (**D**) Height profiles along the white lines in C.

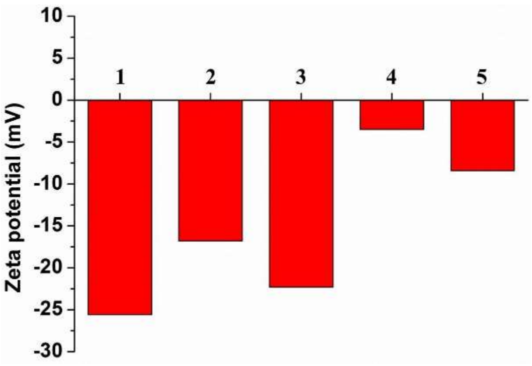

Figure 3. Zeta potentials of BPQDs-based NPs. 1, 2, 3, 4 and 5 represent BPQDs, BPQDs-PEG, BPQDs-PEG-FA, BPQDs-PEG/DOX and BPQDs-PEG-FA/DOX, respectively.

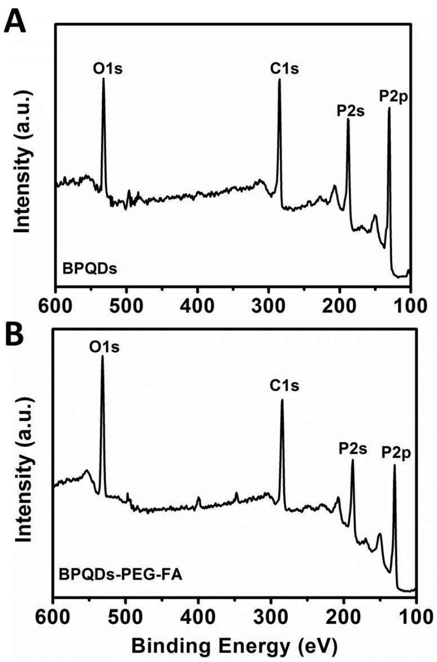

Figure 4. XPS spectrum of (A) BPQDs and (B) BPQDs-PEG-FA.

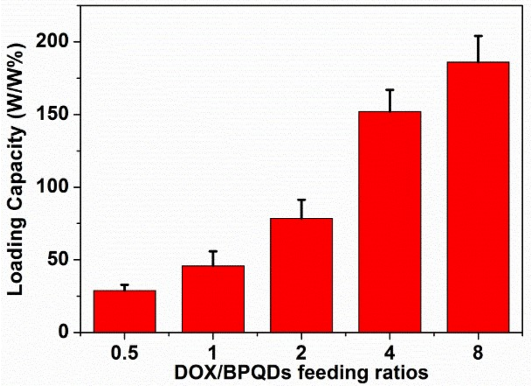

Figure 5. DOX loading capacities on BPQDs-PEG-FA (w/w %) with increasing DOX/NPs feeding ratios.

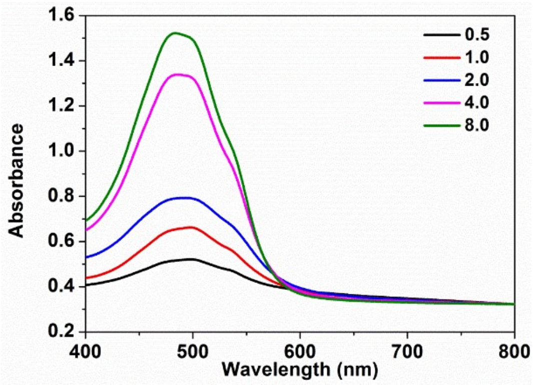

Figure 6. UV–vis–NIR spectra of BPQDs-PEG-FA /DOX at different DOX/NPs feeding ratios after the removal of excess free DOX.

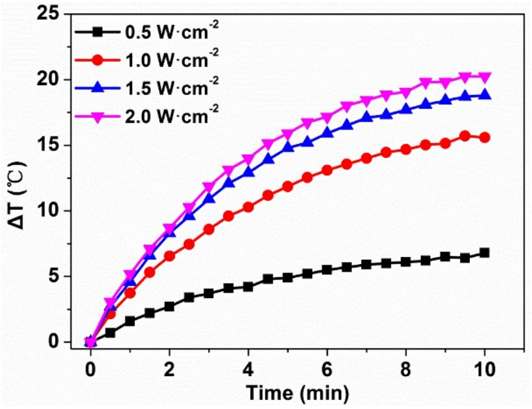

Figure 7. Photothermal heating curves of BPQDs-PEG-FA/DOX under different power intensities.

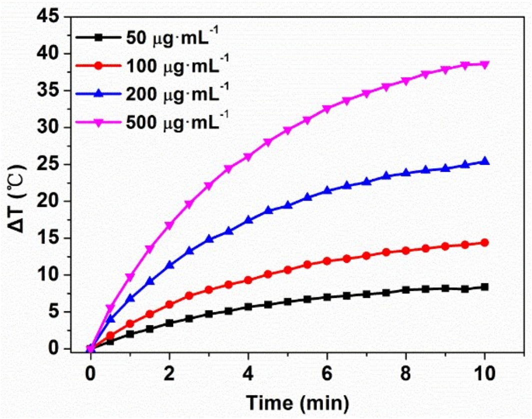

Figure 8. Temperature variation curves of the BPQDs solution under different concentrations (1 W cm^{-2}, 10 min).

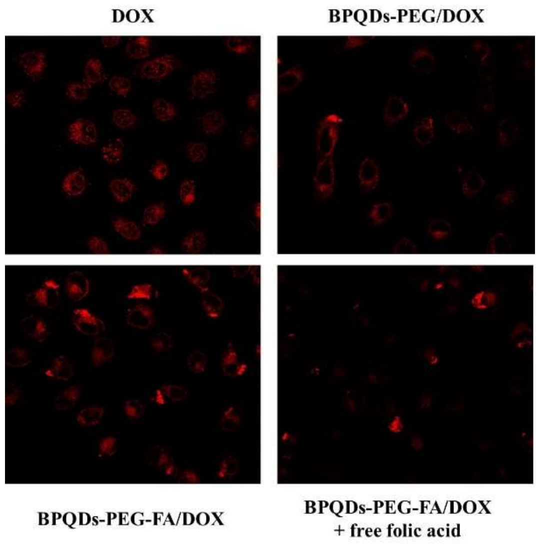

Figure 9. Confocal images of HeLa cells incubated with free DOX, BPQDs-PEG/DOX, BPQDs-PEG-FA/DOX and BPQDs-PEG-FA/DOX + free folic acid after incubation for 0.5 h.

© 2019 by the authors. Submitted for possible open access publication under the terms and conditions of the Creative Commons Attribution (CC BY) license (http://creativecommons.org/licenses/by/4.0/).
